# Disparities in Responsibility Sharing and Gender Differences in Diabetes Care: Changes in Occupational Life of Parents of Children with Type 1 Diabetes

**DOI:** 10.1155/2023/7166604

**Published:** 2023-05-30

**Authors:** Kagan E. Karakus, Sibel Sakarya, Heike Saßmann, Ruken Yıldırım, Şervan Özalkak, Mehmet N. Özbek, Nurdan Yıldırım, Gülcan Delibağ, Beray S. Eklioğlu, Belma Haliloğlu, Murat Aydın, Heves Kırmızıbekmez, Tuğba Gökçe, Ecem Can, Elif Eviz, Gul Yesiltepe-Mutlu, Şükrü Hatun

**Affiliations:** ^1^School of Medicine, Koc University, Istanbul, Turkey; ^2^School of Medicine, Department of Public Health, Koc University, Istanbul, Turkey; ^3^Medical Psychology, Hannover Medical School, Hannover, Germany; ^4^Department of Pediatric Endocrinology and Diabetes, Diyarbakir Child Diseases Hospital, Diyarbakir, Turkey; ^5^Department of Pediatric Endocrinology and Diabetes, University of Health Sciences Gazi Yasargil Training and Research Hospital, Diyarbakir, Turkey; ^6^Department of Pediatric Endocrinology and Diabetes, Dr. Sami Ulus Child Health and Diseases Training and Research Hospital, Ankara, Turkey; ^7^Department of Pediatric Endocrinology and Diabetes, Cukurova University Balcali Hospital, Cukurova University, Adana, Turkey; ^8^Department of Pediatric Endocrinology and Diabetes, Necmettin Erbakan University, Konya, Turkey; ^9^Department of Pediatric Endocrinology, Marmara University, Istanbul, Turkey; ^10^Department of Pediatric Endocrinology, OndokuzMayis University, Samsun, Turkey; ^11^Department of Pediatric Endocrinology and Diabetes, University of Health Sciences Umraniye Training and Research Hospital, Istanbul, Turkey; ^12^School of Medicine, Department of Pediatric Endocrinology and Diabetes, Koc University, Istanbul, Turkey

## Abstract

**Aims:**

To evaluate responsibility sharing between parents of children with type 1 diabetes and change in their occupational status one year after the diagnosis.

**Methods:**

In this cross-sectional multicenter study, parents of children under the age of 18 with a diagnosis of type 1 diabetes answered a questionnaire assessing diabetes-related responsibility sharing between parents, and occupational changes due to child's diabetes. Changes in the occupational status with associated factors and distribution of diabetes-related responsibilities between parents were analyzed.

**Results:**

Among parents of 882 children (mean (SD) age at diagnosis was 7 (3.8) years, female 52.5%), unemployment increased significantly in mothers (59.0% vs. 67.1%; *p* < 0.001), but not in fathers (10.4% vs. 10.7%; *p* > 0.05) within 1 year after their child's diagnosis. Working mother's occupational withdrawal was associated with the child's age at diagnosis (OR = 0.92, [95% CI 0.86–0.99]; *p*=0.02) and mother's education (compared to a university degree or above, high school graduate (OR = 2.93, [95% CI 1.59–5.4]; *p* < 0.001) and not graduated high school (OR = 8.4, [95% CI 3.56–19.83]; *p* < 0.001)). According to the mothers, none of the responsibilities in diabetes care were shared equally between parents, while fathers reported most responsibilities were shared equally. Compared to mothers who preserved their occupation after the diagnosis, mothers who quit their occupation had significantly higher responsibility scores (2.04 vs. 1.55; *p*=0.04), especially in diabetes care at school (*p* < 0.01).

**Conclusions:**

The difference in parents' perceptions of their involvement in their child's diabetes is remarkable. Gender differences in the child's diabetes care extend to the occupational life of parents unequally.

## 1. Introduction

The American Diabetes Association recommends regular glucose monitoring, insulin administration, nutritional planning, physical activity, and psychological and social support for optimal management of type 1 diabetes in children [1]. This intensive and comprehensive management places a significant burden on caregivers, particularly for children under the age of six who are less self-sufficient and have more challenging glucose management needs [2–4].

The daily routines of most families undergo radical changes due to the burden of type 1 diabetes. Most families hesitate to entrust their child to others, wait at school for meals and injections [4], have impulses to constantly monitor their children and their glucose, and sleep less [5]. Additionally, parents are at risk of depression due to chronic anxiety and exhaustion [6]. The effects of a child's diabetes also extend to the social and professional lives of parents who may consider leaving their jobs to care for their child [2, 7]. Thus, sharing the responsibility is crucial in reducing anxiety and anger, preventing depression, easing the child's care, and achieving better glycemic control [8–10].

In this regard, the distribution of diabetes-related responsibilities between parents is not well studied and the studies on the parents' occupational life after a child's diagnosis are limited. Thus, we investigated the responsibility sharing between parents and its association with the occupational status of parents one year after the child's diagnosis of type 1 diabetes.

## 2. Methods

### 2.1. Settings

This cross-sectional multicenter study was conducted at nine pediatric endocrinology centers in Turkey and at the online network of the Children Diabetes Foundation of Turkey between February 2022 and August 2022. The Ethical Committee at Koç University approved the study procedures (2022.378.IRB3.176) as per the Declaration of Helsinki.

### 2.2. Participants

Parents of children and adolescents with type 1 diabetes were recruited at nine pediatric endocrinology centers. The parents were informed at the clinics during their routine visits about the online questionnaire and anonymous data collection. The online survey link was shared with the parent who was going to fill out the survey. In addition to the clinic participants, the online survey link was distributed to parents through the Children Diabetes Foundation's social media groups twice in June 2022. Online survey responses were collected for all participants via Qualtrics Survey Tool (Provo, UT). Families with more than one child with type 1 diabetes were asked to answer the questions by considering the younger child with type 1 diabetes. Of participants, mothers or fathers whose children were diagnosed before the age of 18 were included. Children with diabetes duration of less than 3 months were excluded to ensure sufficient diabetes experience of families. Caregivers other than the mother or father were excluded since the responsibility distribution and occupational status were compared only between parents.

### 2.3. Questionnaire

Our questionnaire consisted of three parts. The first two parts of the questionnaire were translated from a study upon the author's approval [7]. Accordingly, the first part of the questionnaire covers the child's sociodemographic and clinical characteristics, while the second part includes parents' sociodemographic characteristics, employment statuses before and within 1 year after the diagnosis, and the financial burden of diabetes. The last part of the questionnaire, developed by the authors, includes two questions regarding diabetes care. The first question is about the responding parent's perception of taking responsibility for diabetes care (ranging between 1: equal distribution and 4: only one parent taking all the responsibilities). The second question is about the distribution of responsibilities of diabetes care among parents according to 9 different topics, which are diabetes management at night, at school, mental and emotional challenges, diet, doctor follow-ups, purchasing diabetes equipments, injections/fingersticks, following advancements and decision-making. The parents who filled out the questionnaire answered about their children in the first part of the questionnaire, about both parents in the second part (for example, the mother reported her and the father's employment status), and only about themselves in the third part. The third part of the questionnaire was used here for the first time and was not validated. The questionnaire is presented in supplementary file 1.

### 2.4. Sample Size and Statistical Analyses

The number of people under the age of 20 with type 1 diabetes was estimated as 28454 in Turkey in 2021 [11]. During the six-month period, 1254 people filled out the online questionnaire; after data cleaning, 882 participants (parents) were included in the data analysis. Continuous data were presented as mean and standard deviation (SD), and categorical data were presented as absolute numbers and percentages. The chi-square test was performed to compare categorical variables. To analyze occupational withdrawal, McNemar's test was performed to compare occupation status before and after the diagnosis (full-time and part-time/marginal vs. no employment).

Regardless of marital status, a logistic regression test was performed with previously working mothers' occupation change (“no change” vs. “reduced work”) as the dependent variable and the child's age at the diagnosis, the number of siblings, living arrangement (living with both parents and living with a single parent), mother's education, household income, and socioeconomic ranking of family's provinces as the independent variable. Maternal education was categorized into three groups as less than high school graduate (no education, elementary school graduate, or middle school graduate), high school graduate (high school graduate or apprenticeship/traineeship school graduate), and a university degree or above. The Turkish Ministry of Industry and Technology conducted a “socioeconomic development ranking of provinces research” and ranked 81 provinces in Turkey into 6 groups based on variables such as demographics, employment, education, health, finance, accessibility, and quality of life [12]. The provinces of families were assigned to groups based on this ranking.

Regarding the perception of mothers and fathers who live with a partner on diabetes-responsibility distribution in 9 different topics, the Wilcoxon signed-rank test was used to determine if the median value of responsibility score of the responding parent was equal to the standard value of 5, which indicated an equal distribution on a 11-point scale (0: the mother has all responsibility and 10: the father has all responsibility). The median test was used to compare the median values between the mother and father responders and between mothers who quit their occupations and mothers who preserved their occupations. The same test was also used for comparing the median values of mothers and fathers by diabetes technology (pump and/or continuous glucose monitoring) use. The scores of 9 responsibility topics summed to produce a combined responsibility score, treated as a continuous variable, and compared with the *T*-test between mothers who quit their jobs and who preserved their occupations. IBM SPSS v28.0 software was used for statistical analysis. The threshold for statistical significance was two-sided *p* < 0.05.

## 3. Results

### 3.1. Study Population

A total of 1254 responses were collected in the study; 372 responses were excluded due to missing information or duplicate responses. Among the final 882 responses, 692 were from 9 pediatric endocrinology clinics, and 190 were from the online network of the Children Diabetes Foundation. Participants were from 65 out of 81 provinces of Turkey. According to the socioeconomic development ranking of the provinces, four of the 9 diabetes centers were located in the first region (socioeconomically most developed), one in the second, two in the third, and two in the sixth region (socioeconomically least developed).

Participants' characteristics are shown in Table 1. Most responders were mothers (77.9%), and all responders answered for the entire family except the questions about responsibility distribution. The children's mean (SD) age at the diagnosis was 7 (3.8) years, with a mean diabetes duration of 3.7 (3.5) years. Of 882 children, 44.2% were diagnosed before the age of 6, 31.4% between the ages of 6 and 10, and 24.4% between the ages of 11 and 18. Of the children, 829 (94%) were living with both parents, and 53 (6%) were living with a single parent. Among all, 666 (75.5%) children had at least one sibling, 86 (13%) of them had a sibling with a chronic medical condition and 25 families (2.8%) had more than one child with type 1 diabetes.

While 16.3% of responders did not know the child's last HbA1c value, father responders significantly knew less than mothers (79% vs. 85%, respectively; *p*=0.04). According to self-reported 738 HbA1c values, the mean (SD) of the last HbA1c was 7.5% (58 mmol/mol) (1.4). The reported current pump and CGM users were 171 (19.4%) and 438 (49.7%), respectively.

### 3.2. Parent's Occupational Status before and within One Year after the Diabetes Diagnosis

Among the 882 parents, 72 parents who reported a change in occupation status for reasons other than their child's diabetes diagnosis were excluded from the following analysis.

**Figure 1:**
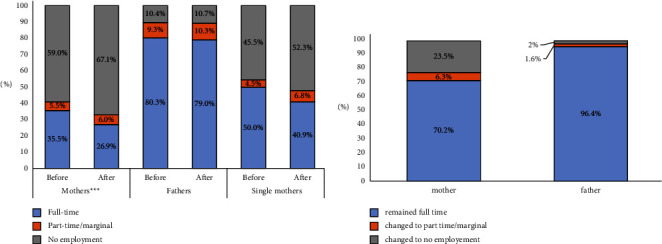
(a) Employment status of mothers who live with their partner (*n* = 766), fathers who live with their partner (*n* = 766), and single mothers (*n* = 44) before and in 1 year after diabetes diagnosis. Reasons for unemployment of mothers were reported with proportions to the total unemployment before and after diabetes diagnosis, respectively, as follows: on maternal leave (0.3 to 3.1%), retired (0.8 to 0.9%), looking for work (11.0 to 9.3%), unspecified (1.0 to 1.0%), and being a homemaker (46.0 to 52.9%). Reasons for unemployment of fathers were reported with proportions to the total unemployment before and after diabetes diagnosis, respectively, as follows: retired (2.1 to 2.3%), looking for work (5.7 to 6%), and unspecified (2.6 to 2.4%). Change in occupational status was significant at ^*∗∗∗*^*p* < 0.001 according to McNemar test. (b) Employment status change of previously full-time working mothers who live with their partner (*n* = 272) and fathers who live with their partner (*n* = 615) in 1 year after the diagnosis.

Among 766 partners who live together, the full-time employment of mothers decreased from 35.5% before the diagnosis to 26.9% the year after. Part-time/marginal employed mothers increased from 5.5% to 6.0%, and unemployed mothers increased from 59.0% to 67.1% (Figure 1(a)). McNemar's test showed that formerly working mothers living with a partner have withdrawn significantly from their occupations within 1 year of the diagnosis (*p* < 0.001). Of 314 previously employed mothers, 26.4% stopped working, and 6.3% reduced working hours. Among 272 previously full-time employed mothers with partners, 23.5% quit their jobs, and 6.3% changed their working status to part-time or marginal employment (Figure 1(b)). Among 766 partners who live together, the full-time employment of fathers decreased from 80.3% before the diagnosis to 79% the year after. Part-time/marginal employed and unemployed fathers increased from 9.3% to 10.3% and from 10.4% to 10.7%, respectively (Figure 1(a)). McNemar's test demonstrated that fathers have not withdrawn from occupational life significantly (*p*=0.864). Among 686 previously working fathers, 2.6% stopped working, and 1.6% reduced working hours. Among 615 previously full-time working fathers, 2% stopped working, and 1.6% changed their working status to part-time or marginal employment (Figure 1(b)).

Regarding 44 single mothers, the percentage of full-time employment reduced from 50.0% to 40.9%, while part-time employed and unemployed single mothers increased from 4.5% to 6.8% and 45.5% to 52.3%, respectively (Figure 1(a)). However, single mothers' withdrawal from occupational life was not significant (*p*=0.375).

Logistic regression analysis was performed to find the associated factors for occupational status change (no change vs. reduced work) in 335 previously working mothers regardless of marital status. The quitting or reducing work was significantly associated with the child's age at the diagnosis (OR = 0.92 [95% CI 0.86–0.99]; *p*=0.02) and the mother's education. The odds for quitting or reducing work were higher in high school graduate mothers (OR = 2.93 [95% CI 1.59–5.4]; *p* < 0.001) and mothers who did not graduate high school (OR = 8.4 [95% CI 3.56–19.83]; *p* < 0.001), compared to mothers with a university degree or above.

**Figure 2:**
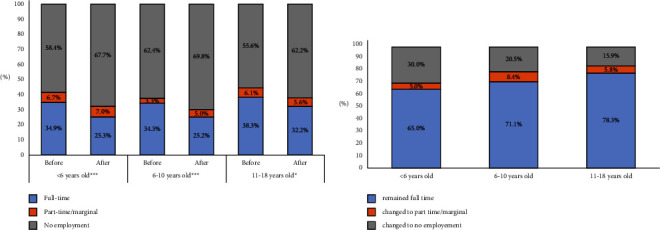
(a) Employment status of mothers who live with a partner before and after the diagnosis by the child's age at the diagnosis (*n* = 766). <6 years *n* = 344, 6–10 years *n* = 242, and 11–18 years *n* = 180. (b) Employment status change of previously full-time working mothers with a partner (*n* = 272) in 1 year after the diagnosis by the child's age at the diagnosis. Children's ages are grouped into three categories: <6 years (*n* = 120), 6–10 years (*n* = 83), and 11–18 years (*n* = 69). Change in occupational status was significant at ^*∗∗∗*^*p* < 0.001 and ^*∗*^*p* < 0.05 according to McNemar test.

As shown in Figure 2(a), occupational statuses have changed for mothers in all groups by child's age at the diagnosis (*p* < 0.001 for age <6 and age 6–10, *p*=0.023 for age 11–18). Among full-time working mothers, 78.3% of those whose child was older than 10 at the time of diagnosis continued to work full-time; this rate dropped to 71.1% in the 6–10 age group and to 68% under the age of 6 (Figure 2(b)).

**Figure 3:**
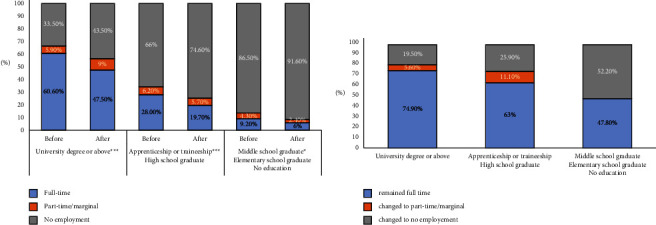
(a): Mothers' occupational status change by mother's education. Education levels are grouped into three categories: university degree or above (*n* = 322), high school or apprenticeship/traineeship graduates (*n* = 193), and middle school graduate, elementary school graduate, or no education (*n* = 251). (b) Employment status change of previously full-time working mothers in 1 year after the diagnosis. University degree or above (*n* = 195), had high school degree or apprenticeship/traineeship (*n* = 54), and had middle or elementary school degree or no education (*n* = 23). Change in occupational status was significant at ^*∗∗∗*^*p* < 0.001 and ^*∗*^*p* < 0.05 according to McNemar test.

Figure 3(a) shows the distribution of employment status of women by education level. Accordingly, higher education was associated with more employment (*p* < 0.001) (Figure 3(a)). Furthermore, higher education was associated with less withdrawal from employment after the diagnosis (*p* < 0.001), as shown in Figure 3(b).

When the financial burden of diabetes was asked, 12.8% of respondents reported no loss to few financial loss, 29.8% moderate loss, and 57.4% high to severe financial loss.

### 3.3. Distribution of Responsibilities among Parents and Their Perspectives

Question about the distribution of responsibilities in diabetes care was answered by 754 parents who live with a partner (576 mothers and 178 fathers) and all responders answered according to their own perspectives and not answered behalf of their partners. While 32.1% of responding parents reported that the responsibilities were shared equally, 24.7% reported that they took a little more responsibilities, 20.5% took most of the responsibilities, and 22.7% took all of the responsibilities. When the answers to this question were compared by gender, the proportion of “taking all or most of the responsibility” differed significantly (54% in mothers vs. 8.4% in fathers; *p* < 0.001).

**Figure 4:**
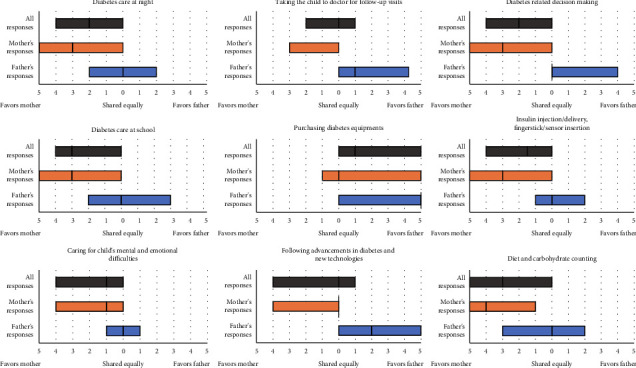
Distribution of diabetes-related responsibilities among parents who live together. Of all responders (grey boxes, *n* = 754), 564 were mothers (orange boxes) and 178 were fathers (blue boxes). Boxes represent interquartile ranges while the median was shown as vertical line in the boxes. Whiskers are not shown since the range is 0, and 10 for all.

Responding parents' perceptions of their role in diabetes care were questioned on 9 topics, as shown in Figure 4. Accordingly, the distribution of responsibility differed significantly between mother and father responders in all topics (*p* < 0.001; for all topics), reflecting disparities in the perceptions of mothers and fathers.

One-sample Wilcoxon signed-rank test was performed to determine if the responses represent an equal sharing of responsibility for all responders, mother responders, and father responders. According to the mothers, all responsibilities were shared unequally (*p* < 0.001 for all topics) and all responsibilities were firmly on the mothers except for purchasing diabetes equipment, which was the responsibility of the fathers. According to the father responders, the responsibility of purchasing diabetes equipment was firmly on fathers (*p* < 0.001). However, the child's mental and emotional difficulties, diabetes management at school, management at night, insulin injections/fingerstick/sensor insertions, and diet and carb counting were not shared unequally (*p* > 0.05), reflecting an equal distribution of these responsibilities between parents in the fathers' perspective.

The mean responsibility score of 9 topics was significantly higher in mothers who quit their work than in mothers who preserved their occupational status (2.04 vs. 1.55; *p*=0.04). Among previously working mothers, mothers who quit their work reported significantly more responsibility only in diabetes care at school (*p* < 0.01) than mothers who preserved their occupational status.

Among mother respondents, only the responsibility in “diabetes care at night” was significantly differed (*p* < 0.001) by technology use (pump and/or CGM use) stating technology use provided more equal responsibility distribution between parents at night. Among father respondents, there was not significant change in responsibility distribution between parents by technology use.

## 4. Discussion

This study investigated disparities in responsibility sharing between parents and changes in the occupational life of parents after their child's type 1 diabetes diagnosis.

Our study demonstrated that mothers sacrifice their occupations while undertaking more responsibility for their children's diabetes. The higher unemployment rates of mothers than fathers show an already well-established disparity of participation in work life both before and after the diagnosis in Turkey [13]. However, the diagnosis further exacerbates this disparity and places mothers in a more disadvantaged position in a professional life. In contrast, the unemployment rate among fathers was not affected by the diagnosis and remained similar to the national unemployment rate [13]. Single mothers had higher participation in occupational life and less withdrawal than mothers with a partner. Economic support from a partner might facilitate the withdrawal of mothers from occupational life.

A study with 134 parents, reported a reduction and cessation of occupation of mothers of children with type 1 diabetes in the United States [2]. Two studies from Germany showed that mothers reduced their working hours significantly after the diagnosis, which was not significant in fathers [7, 14]. To our knowledge, this is the first study from a developing country that investigated the occupational changes of parents after their child was diagnosed with type 1 diabetes. Furthermore, our results can be compared with the recent study from Germany [7] as the same employment assessment questionnaire was used. The mothers' transition from full-time to part-time/marginal work was higher in Germany than that in our results (25.3% vs. 6.3%), while most of the mothers in Turkey withdrew entirely from their work life as they quit their full-time occupation. The unemployment rates among mothers differed between the two studies (59% in Turkey vs. 25.8% in Germany). Thus, mothers in Turkey were more prone to disconnect from occupational life. Possible explanations for these findings may be fewer opportunities to work part-time for Turkish mothers or more social pressure to stay at home. While the proportion of women working part-time in employment is 19.5% in Turkey and 9.3% in men [15], these rates are 47% and 11%, respectively, in Germany [16].

A child's younger age at the diagnosis was a risk factor for the mother's withdrawal from work life in our study, similar to the studies from Germany [7, 14]. However, mothers' education was also a significant risk factor for withdrawal in our study. Mothers with higher education were less withdrawn from their work life, which also shows that education increases mothers' participation and strengthens the mother's place in work life. In our study, the occupational participation of women before the diagnosis is consistent with the data from the Turkish Statistical Institution [15]. In 2020, 66% of the university-educated women and 30% of the high school graduates in Turkey participated in the labor force, while in our study the participations were 67% and 34%, respectively [15]. In this regard, our study participants represent working women in Turkey to some extent. This suggests that the diagnosis of type 1 diabetes leads to mothers leaving the workforce at all education levels in Turkey, and further research should focus on what is needed to help mothers to stay occupied.

We showed that mothers carry most of the burden of diabetes, and they do more strenuous work such as diabetes care at school, which affects their professional life. On the other hand, the working status of fathers is less affected. This shows the effect of gender roles and it can be speculated that diabetes care is perceived as a duty of women such as “housework.” According to a study from Turkey, 91% of fathers consider that mothers are the primary caregiver of their children [17]. Lack of fathers' involvement results in unequal distribution of duties within the family, and such inequality leads not only to gender-inequitable role models for children but also to an overall disadvantage for women due to the disproportionate responsibility of care work and household duties. While previous studies showed that mothers have more parental distress and anxiety [6, 8, 18], responsibilities of diabetes affect mothers' lives in the long term, such as problems in their professional life [7, 14, 19], being away from life activities that provide well-being [4, 20], burnout [21], and depression [22–24].

A study conducted on 31 families of children with insulin dependent diabetes aged 12–15 showed that diabetes-related responsibility sharing perceived significantly different between mother and fathers [25]. Similarly, our results demonstrated a significant difference in the perception of burden sharing in a large group of parents of all age groups from young children to late adolescents. Fathers had the perception of “equal” sharing in most subjects, while mothers felt responsible in almost all subjects. This may lead to emerging feelings of mothers such as lack of empathy, “not getting credit for their efforts,” and “feeling alone while dealing with a problem,” in addition to the physical struggle caused by the disparity in burden sharing [26]. Although fathers may support family in different ways such as financial support, planning of daily tasks [26], and the balance, and communication between parents are crucial to prevent isolation and misunderstandings.

Mothers who quit their job take on more responsibilities than fathers and are more burdened with diabetes care at school than working mothers. Entrusting the child, worry about hypoglycemia, and challenging remote glycemia management were reported problems in diabetes management at school previously [27, 28]; however, its effect on parental occupation is shown here for the first time. Responsibilities in this area can be the primary determinant in quitting a job. There has been a national “Diabetes at School Program” in Turkey since 2010, which increased awareness, informed children, teachers, school staff, provided school nurses for children with type 1 diabetes, and improved diabetes care at schools [29]. Expansion and further improvements in the program may reduce the anxiety of families [27, 30], and mothers' withdrawal from professional life.

Although technology use improves and facilitates diabetes care at many aspects such as care at night, at school, or during exercise [27, 31]; responsibility sharing was not affected by technology use except care at night. This indirectly shows that engaging with diabetes technologies is similar with other responsibilities and use of technology did not increase the participation of fathers to the diabetes care from mother's perspective.

In a study from Germany, nearly half of the families (46.4%) stated that diabetes brings a moderate to a severe financial burden. The financial burden was higher in Turkey than that in Germany. The lack of reimbursement for diabetes technologies and difficulties in access can be the main reasons behind this burden [31].

Participation of fathers was relatively less compared to mothers in our study similar to previous studies [7, 32] which showed less involvement of fathers in child's daily health-related issues, presumably due to an existing imbalance in working hours. Furthermore, fathers knew HbA1c values less, which may indirectly show their less involvement in their child's diabetes. Less involvement of fathers leads to less representation, which may create a bias for the studies where the distribution of responsibility was based on self-report. With more father responders, the split between mother and father respondents in the responsibility domains could have been less profound. Further studies should examine barriers to fathers' engagement.

While working on improving the lives of children with type 1 diabetes it should be noted that this chronic multifaceted condition shadows not only the lives of children but also caregivers, especially mothers. Gender roles have a significant effect on sharing the burden of diabetes. To effectively address this problem, a holistic approach in diabetes teams is needed while incorporating psychologists and social workers. To alleviate the burden on mothers, new programs such as “Diabetes at School Program” should be initiated to cover other aspects of lives of families with diabetes and promoting equal responsibility sharing [27, 29, 32].

To our knowledge, this is the first study that demonstrated the perceptions of parents in diabetes-related responsibility sharing, and showing the association between responsibility sharing and change in the occupational status of parents. The large study group from multiple provinces with varying developmental levels was a strength of our study. Another strength was identifying occupational changes caused by diabetes, so only diabetes-related occupational changes were included.

## 5. Limitations

One of the limitations of the study was that we could not do a random sampling, and we received voluntary participation from the families we reached through clinics and NGOs. This may bias the selection of certain families in terms of their ability to participate and respond to the online survey. Our results compared before and within 1 year after the diagnosis and showed the acute impact of diabetes on the employment status. Longitudinal studies are needed to observe the adjustment of families to their new lives with type 1 diabetes beyond 1 year after the diagnosis.

Another limitation was the representation of each family by one parent, using mother and father dyads with random sampling could provide a better comparison and representation of the population. Besides self-reported data on parental involvement in diabetes management, using logbooks for activities such as insulin injection and blood glucose monitoring may provide a more objective methodology for understanding the distribution of responsibility.

## 6. Conclusion

Our study shows the inequality in sharing responsibility between parents while showing the disparity in the perception of mothers and fathers. While mothers report having undertaken more responsibilities for their child's diabetes, fathers think that the tasks were shared equally. A similar disparity was also present in their occupational lives; while mothers quit their works because of the diabetes of their children, fathers' occupational lives were not affected. New initiatives to support mothers and equal distribution of responsibilities among parents can balance this inequality, thus avoiding the disconnection of mothers from their occupational lives.

## Figures and Tables

**Table 1:** Characteristics of the children with type 1 diabetes, mothers, fathers, and the household.

	*N*	*n* (%)
Child		
Current age, median (IQR), years	882	10.6 (7.2–13.8)
Sex	882	
Female		463 (52.5)
Male		419 (47.5)
Diabetes duration, median (IQR), years	882	2.5 (1.1–5.3)
Age at diabetes diagnosis, median (IQR), years	882	6.7 (3.8–9.9)
<6 years		390 (44.2)
6–10 years		277 (31.4)
11–18 years		215 (24.4)
Child's living arrangement	882	
With both parents		829 (94)
With a single mother		51 (5.8)
With a single father		2 (0.2)
Children with sibling(s), no	882	666 (75.5)
Number of siblings, median (IQR)	882	1 (1.0)
Siblings with chronic medical condition	666	86 (13.0)
Siblings with type 1 diabetes	666	25 (2.8)
The last HbA1c, median (IQR), %	738	7.2 (6.5–8.0)
Current use of CGM	882	438 (49.7)
Current use of insulin pump	882	171 (19.4)
Parents (mother/father)		
Completed the questionnaire	882	687 (77.9%)/195 (22.1%)
Age at child's diagnosis, median (IQR), years	881/877	34.9 (30.8–38.9)/38.1 (34.2–42.3)
The latest educational qualification	882/882	
University degree or above		379 (43%)/400 (45.4%)
High school (apprenticeship or traineeship) graduate		65 (7.4%)/84 (9.5%)
High school graduate		156 (17.7%)/171 (19.4%)
Middle school graduate		101 (11.5%)/106 (12%)
Elementary school graduate		125 (14.2%)/108 (12.2%)
No education		56 (6.3%)/14 (1.5%)

*Note*: Percentages were calculated excluding missing values. CGM: continuous glucose monitoring, IQR: interquartile range.

## Data Availability

The data used to support the findings of this study are available from the corresponding author upon request.

## Supplementary Materials

Supplementary Materials Supplementary File 1: questionnaire. Questionnaire used in this study, translated from Turkish to English.
