# Supplementary material for: Disparities in Responsibility Sharing and Gender Differences in Diabetes Care: Changes in Occupational Life of Parents of Children with Type 1 Diabetes
Source: Pediatr Diabetes. 2023 May 30;2023:7166604. doi: 10.1155/2023/7166604 (PMC12017241; doi:10.1155/2023/7166604)
Supplement: Supplementary Materials — Supplementary File 1: questionnaire. Questionnaire used in this study, translated from Turkish to English. [file 7166604.f1.docx]

Responsibility Sharing Among Parents After Diagnosis of Type 1 Diabetes and Its Effect on Work Life

Start of Block: Default Question Block

Dear parents of children with type 1 diabetes,

We know that caring for a child with type 1 diabetes entails many responsibilities and a significant burden every day.

With this questionnaire, we want to learn what kind of consequences it has on parents and family, especially on work life, after your child is diagnosed with type 1 diabetes. We also aim to gather information about what is beneficial for you in your daily life, where you are having difficulties and how the burden is shared within the family.

We want to use the data we obtain from your responses to describe the efforts of parents of children with diabetes, to highlight the need for quality support to policy makers and healthcare providers, and to develop support models.

Your participation is completely voluntary. No data will be collected to identify you or your child. Your participation or non-participation has no positive or negative effect on diabetes treatment.

While filling out the questionnaire, we ask you to mark the most appropriate option for you in multiple choice questions, and to mark the most appropriate point for you by swiping the blue dot on the bar in some graded questions. Directions on the rating bar will help you.

It is sufficient for one of the parents to fill out the questionnaire.

If you want to learn more about the survey, you can contact us by writing to kkarakus16@ku.edu.tr. Thank you in advance for your participation!

| Page Break |  |
| --- | --- |

In which center are you being followed?

- Koç University Hospital (İstanbul)
- Diyarbakır Children’s Hospital (Diyarbakır)
- Diyarbakır Gynecology and Children's Hospital (Diyarbakır)
- Çukurova University Balcalı Hospital (Adana)
- Ondokuz Mayıs University Hospital (Samsun)
- University of Health Sciences Ümraniye Training and Research Hospital (İstanbul)
- Marmara University Pendik Training and Research Hospital (İstanbul)
- Necmettin Erbakan University Hospital (Konya)
- Dr. Sami Ulus Child Health and Diseases Training and Research Hospital (Ankara)
- Other (please specify)__________________________________________________

What is the gender of your child with diabetes:

- Male
- Female

|  |
| --- |

What is the birth date of your child with diabetes?

________________________________________________________________

What type of diabetes does your child have?

- Type 1 Diabetes
- Type 2 Diabetes
- Other type of diabetes

What is the date of diabetes diagnosis?

________________________________________________________________

What treatment is your child currently using for diabetes?

- insulin pen/syringe
- insulin pump

How do you measure glucose?

- Fingersticks/fingerpokes
- Real-time Sensor with alarm (CGM)
- Intermittent Sensor without alarm (FGM)

What is your child's last HbA1c value?

- I know (please specify) ___,___%
- I do not know

Does your child with diabetes have sibling(s)?

- Yes (Please write number of siblings) __________________________________________________
- No

Do any siblings of your child with diabetes have a chronic disease?

- No
- Yes (please specify) __________________________________________________

Who does your child with diabetes live with most of the time? (Please tick one box)

- Together with both parents in one household
- With mother only
- With father only
- Someone else (Please specify) __________________________________________________

| Page Break |  |
| --- | --- |

End of Block: Default Question Block

Start of Block: Block 3

What is the reason why the child with diabetes does not live in the same house with his mother and father?

- Divorce
- Death
- Work-related
- Other(please specify) __________________________________________________

End of Block: Block 3

Start of Block:

THE FOLLOWING QUESTIONS ARE ABOUT YOU AS A PARENT.

Who is completing this survey?

- Mother
- Father
- Other (please specify)__________________________________________________

End of Block:

Start of Block: ebeveyn

Please write the age of the parents and the province they currently live in (if one parent is not alive, you can leave the blank)

- Mother’s age __________________________________________________
- Mother’s province__________________________________________________
- Father’s age__________________________________________________
- Father’s province __________________________________________________

Please select the highest level of education completed for both parents.

|  | Not graduated | Elementary school graduate | Middle school graduate | High school graduate | High school (Apprenticeship or traineeship) graduate | University degree or above |
| --- | --- | --- | --- | --- | --- | --- |
| Mother |  |  |  |  |  |  |
| Father |  |  |  |  |  |  |

Please write clearly below what work both parents **currently** do. If they do not work, mark the relevant box.

|  | If they are working,  please write their job. | Tick if  unemployed/housemaker |
| --- | --- | --- |
| Mother | _____________ |  |
| Father | _____________ |  |

What was the employment status of both parents **before your child was diagnosed** with diabetes? Please specify for both parents

|  | Full-time employment | Part-time employment | Marginal employment (up to 2000 Turkish Lira/month) | Unemployed, wasn't looking for a job | Unemployed and looking for a job | Retired | parental leave | Student | Unable to work due to physical disability or illness |
| --- | --- | --- | --- | --- | --- | --- | --- | --- | --- |
| Mother |  |  |  |  |  |  |  |  |  |
| Father |  |  |  |  |  |  |  |  |  |

What was the employment status of both parents **in the first year after your child was diagnosed** with diabetes? Please specify for both parents.

|  | Full-time employment | Part-time employment | Marginal employment (up to 2000 Turkish Lira/month) | Unemployed, wasn't looking for a job | Unemployed and looking for a job | Retired | parental leave | Student | Unable to work due to physical disability or illness |
| --- | --- | --- | --- | --- | --- | --- | --- | --- | --- |
| Mother |  |  |  |  |  |  |  |  |  |
| Father |  |  |  |  |  |  |  |  |  |

If there was a change in occupation status, was it for a reason other than your child's diabetes diagnosis (such as the Covid epidemic)?

- Yes
- No, It was because of the diabetes diagnosis
- No change in work status

How would you estimate the financial burden your child's diabetes puts on your family? (Please mark only one answer)

- No financial loss
- Minimal financial loss
- Moderate financial loss
- Substantial financial loss
- Severe financial loss

What is the approximate total monthly income of the household? (in Turkish lira) ________________________________________________________________

| Page Break |  |
| --- | --- |

To what extent are the responsibilities for your child's diabetes care shared between parents? Please tick the option most suitable for you.

- I take all the responsibilities, I bear all the burden
- I do most of the responsibilities
- Although we share responsibilities, I take on a little more
- As parents, we share the responsibilities equally as our daily life allows.

In general, how are the responsibilities for your child's diabetes care shared between parents? Please move the blue dots on all the bars to the place you want for each sentence. (0: mother takes all responsibility, 5: responsibility shared equally, 10: father takes all responsibility)

|  | **Mother does** | **Equally shared** | **Father does** |
| --- | --- | --- | --- |

|  | 0 | 1 | 2 | 3 | 4 | 5 | 6 | 7 | 8 | 9 | 10 |
| --- | --- | --- | --- | --- | --- | --- | --- | --- | --- | --- | --- |

| Diabetes care at night | 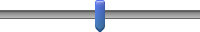 |
| --- | --- |
| Taking the child to the doctor for routine diabetes control | 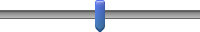 |
| Diabetes care at school | 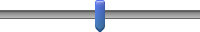 |
| Adjusting diet and counting carbohydrates | 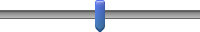 |
| Handling with the child's mental difficulties and emotional problems | 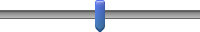 |
| Deciding insulin doses and correction doses, following the glucose course | 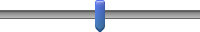 |
| Injecting/delivering insulin, measuring glucose | 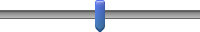 |
| Purchasing diabetes-related materials (insulin, sensors, etc.) | 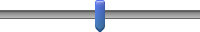 |
| To follow current developments and technologies | 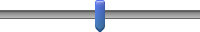 |

End of Block: ebeveyn
